# Driven by Dopamine: Genetic Insights into Motivation and Performance in Sports and Esports

**DOI:** 10.3390/genes17020144

**Published:** 2026-01-28

**Authors:** Natalia Majchrzak, Kinga Humińska-Lisowska, Agata Leońska-Duniec

**Affiliations:** 1 Doctoral School, Faculty of Physical Education, Gdansk University of Physical Education and Sport, 80-336 Gdansk, Poland; natalia.majchrzak@awf.gda.pl; 2 Faculty of Physical Education, Gdansk University of Physical Education and Sport, 80-336 Gdansk, Poland; kinga.huminska-lisowska@awf.gda.pl

**Keywords:** dopaminergic system, genetic polymorphisms, *DRD2*, *DRD4*, *COMT* Val158Met, esports performance

## Abstract

**Background/Objectives**: The dopaminergic system regulates motivation, executive functions, motor learning, and emotional responses—processes that are key in both sport and esports. Although many studies analyse dopaminergic gene polymorphisms, their impact on psychophysical predispositions remains unclear. This narrative review aims to summarise current knowledge about the mechanisms of dopamine action and genetic determinants that may influence athletic and cognitive performance. **Methods**: The PubMed, Scopus, and Web of Science databases (publications from January 2010 to December 2025) were searched using keywords related to the *DRD1–DRD5*, *COMT*, *SLC6A3/DAT1*, and *TH* genes, as well as the terms ‘sport’ and ‘esport.’ Studies of athletes were included in which the relationship between dopaminergic polymorphisms and motivational and personality traits was assessed, and the results of neuroimaging and epigenetic studies were also considered. **Results**: Dopaminergic polymorphisms are associated with differences in reward processing, cognitive flexibility, motivation, and stress resilience. The most essential critical effects concern the *DRD2* and *DRD4* variants, which are associated with novelty seeking, reward dependence, and coping with stress. The *COMT* Val158Met polymorphism affects dopamine levels in the prefrontal cortex, modulating executive functions. The effects of individual polymorphisms are moderate, and conclusions regarding esports remain speculative due to limited research in this area. **Conclusions**: Dopaminergic predispositions involve interactions among genetics, neural activity, and the environment. However, current evidence is limited by small sample sizes, a predominance of European populations, scarce data on esports players, and difficulties in separating genetic effects from training-related adaptations.

## 1. Introduction

Since Galton’s time (1875), twin studies have become a fundamental approach for analysing the contributions of genetic and environmental factors, with study design expanding to include both monozygotic and dizygotic twins, as well as multigenerational and adoptive families. Early twin studies indicated a significant influence of genetics on behaviour but did not allow for the identification of specific genetic variants. Only later approaches, integrating epigenetics and gene-environment interactions, demonstrated that even monozygotic twins differ in methylation patterns and gene expression, considerably broadening the interpretation of classical twin studies [1,2]; interpretations based solely on early twin designs were, therefore, partly limited and prone to systematic error.

Developments in molecular biology and functional genetics have enabled more precise investigation of specific gene variants, including those related to the dopaminergic system, which plays a central role in the regulation of cognitive, motivational, and emotional functions. These mechanisms are particularly important in the context of sport, including traditional disciplines: intense physical exertion and training are associated with increased dopaminergic activity and improvements in cognitive flexibility, learning speed, and tactical adaptation in athletes [3]. This review adopts a conceptual framework in which dopaminergic gene variability is proposed to partly modulate neural signalling, contributing to inter-individual differences in motivational and personality traits (e.g., novelty seeking and stress resilience). These genetic factors are recognised as influencing behaviours related to performance in sport and esports, as part of a broader, multifactorial system that also includes training conditions and experience. In esports, increasingly seen as an activity requiring high mental and physical fitness, digital competition involves complex processes such as selective and divided attention, reaction speed, and emotional control under time pressure [4,5]. Furthermore, meta-analyses indicate that regular play of action games can lead to improved stimulus processing, working memory, and cognitive flexibility [6,7,8,9]. However, direct genetic evidence in esports remains scarce, and most current hypotheses are extrapolated from studies conducted in traditional sports or related cognitive domains. This raises the fundamental question of whether dopaminergic gene polymorphisms, associated with motivation, stress resilience, and executive function in athletes, may also be relevant for performance in esports players.

The aim of this review is therefore to present the current state of knowledge on the genes of the dopaminergic system and their potential links to psychophysical predispositions in both athletes and participants in digital competition. This study refers to a narrative review, which emphasises the importance of integrating genetic and epigenetic research in sport and esports [3]. Based on available empirical evidence, this review discusses the mechanisms of dopamine action, receptor function, and the role of selected genetic polymorphisms in shaping traits relevant to physical performance, cognitive ability, and psychological adaptation to stress.

## 2. Materials and Methods: Narrative Review Design

This review was narrative in nature, which distinguishes it from a systematic review primarily in terms of the flexibility of literature selection and data interpretation. As Sukhera notes, narrative reviews allow for the synthesis of studies employing diverse methodologies and provide a critical analysis of the current state of knowledge, with their main value lying in the integration of multiple perspectives [10]. For this reason, despite the lack of a formal protocol registration, the review followed predefined eligibility criteria and transparent procedures for literature identification, selection, and synthesis, with an emphasis on methodological rigour.

The following electronic databases were searched: PubMed, Scopus, and Web of Science. Following the example of studies on the genetic determinants of aerobic capacity [11], a broad time frame was applied, covering publications from 1 January 2010 to 31 December 2025. Selected seminal studies published before 2010 were additionally included to provide essential theoretical and neurobiological background on dopaminergic signalling mechanisms; however, these works were not part of the primary literature screening and were cited solely to support conceptual frameworks and well-established biological principles. No conclusions regarding sport or esports-related genetic associations were drawn exclusively from studies published before 2010. The inclusion and exclusion criteria, as well as the time frame of the literature search, were defined before the screening process was initiated.

The literature search employed combinations of keywords related to dopaminergic genes (e.g., “*DRD1*”, “*DRD2*”, “*DRD3*”, “*DRD4*”, “*DRD5*”, “*COMT*”, “*SLC6A3/DAT1*”, “tyrosine hydroxylase”) and terms describing performance domains (“sport”, “athletics”, “esports”), psychological constructs (“motivation”, “cognitive function”, “executive functions”, “reward processing”), as well as “polymorphism”, “SNP”, “gene polymorphism” and “epigenetics”. Keywords were combined using AND/OR operators.

Original articles, reviews and meta-analyses meeting the following criteria were included: (1) the study involved humans; (2) the relationship between dopaminergic gene polymorphisms and sports performance, personality traits, motivation, executive functions or training adaptation was analysed; (3) the participants were amateur or professional athletes, or possibly esports players; (4) the article was published in a peer-reviewed scientific journal in English. Due to the limited number of available studies, studies involving non-athletes were also included, provided that the results referred to psychological traits relevant to sport. Studies using only animal models, case reports, letters to the editor, conference abstracts, and articles without full text in English were excluded. In cases where the abstract was in another language, machine translations were used for preliminary assessment, and the final decision was made after analysing the full text.

The selection process was multi-stage. In the first stage, two independent authors reviewed the titles and abstracts of the identified articles, eliminating those that were clearly unrelated to the topic. Publications whose nature was unclear were referred for full-text analysis. Discrepancies in qualification were resolved through discussion; if necessary, a third reviewer was included to reduce bias and increase the reliability of the selection. Information on sample size, gender, age, and ethnicity of participants, type of sport, analysed polymorphisms, psychometric tools, and results was extracted from each article. Data on study design (cross-sectional, cohort, case–control), genotyping methods, and statistical analyses were also included. When contradictory findings were identified, their interpretation considered sample size, population characteristics, replication status, and overall methodological quality. Greater weight was given to findings supported by independent replication or derived from larger and methodologically robust cohorts.

The quality of individual studies was assessed using a modified scale proposed by Hennis et al. and Clark and Baudouin, which was adapted in the review by Bıçakçı et al. [11]. This scale takes into account, among other things, the size and nature of the control group, compliance with Hardy–Weinberg equilibrium, clarity of case group definitions, details of primers used, repeatability of genotyping, blinding of analysts, statistical power calculations, and the validity of analytical methods used. Only studies that met the minimum quality requirements were discussed in detail; in the case of serious methodological shortcomings, the results were interpreted cautiously or excluded from detailed discussion.

The final synthesis was qualitative in nature. Due to the heterogeneity of the measurements used (different psychological tests, various sports disciplines, diverse populations), no meta-analysis was performed. The results were presented in a descriptive form, comparing the effects of individual polymorphisms on psychological traits and sports performance, and identifying recurring patterns and research gaps. Where possible, potential gene-environment interactions, epigenetic mechanisms of gene expression regulation, and neuroimaging results were indicated. This review follows the tradition of narrative reviews, which, although less structured than systematic reviews, allow for a comprehensive analysis and interpretation of complex issues [10], offering the reader an in-depth insight into the role of the dopaminergic system in sports and esports.

## 3. The Dopaminergic System and Its Biological Significance

Dopamine has a number of functions in the brain beyond traditional motor control. Its activity operates in two complementary modes, tonic (baseline) and phasic (burst-like), and plays a central role in the regulation of motor, motivational, and cognitive processes. In general terms, dopaminergic neurons encode information related to reward and behavioural relevance of stimuli, thereby supporting learning, attention, and adaptive behaviour in changing environmental conditions [12,13,14,15,16].

In contrast, dopamine also contributes to motor learning by modulating neural activity involved in the acquisition and refinement of motor patterns. Experimental and neuroimaging studies suggest that dopaminergic signalling supports the adaptation and stabilization of motor behaviour, through its influence on synaptic plasticity, although in humans these effects appear moderate and show limited replicability, partly due to methodological heterogeneity and reliance on animal models [15,17,18].

Another important aspect of dopamine’s action is its involvement in cognitive processes, especially in the regulation of executive functions such as working memory, attentional control, planning and cost–benefit-based decision-making [19]. Dopaminergic activity within the prefrontal cortex, particularly along the mesocorticolimbic pathway, plays a key role in modulating these functions and supporting flexible, goal-directed behaviour [20]. Alterations in dopaminergic signalling, either insufficient or excessive, are associated with suboptimal cognitive performance, highlighting the importance of balanced dopamine regulation for executive functioning [18].

The topography of dopamine action in the brain is based on the functioning of four major dopaminergic pathways: nigrostriatal, mesolimbic, mesocortical, and tuberoinfundibular. Although their anatomical pathways have been well described, the functional specificity of each pathway continues to raise research questions, especially in the context of complex motor, cognitive, and emotional behaviour. The nigrostriatal pathway, running from the substantia nigra (SN) to the striatum, plays a crucial role in motor control, particularly in the initiation and smooth switching of motor sequences. Degeneration of neurons within this pathway leads to the classic symptoms of Parkinson’s disease, such as bradykinesia, resting tremor or muscle rigidity, as confirmed by both neuropathological and neurochemical studies [21,22]. In the context of sport and esports, the integrity of this pathway may be important not only for movement precision and reaction speed, but also for the inhibition of motor automatisms under dynamic task conditions; however, empirical data in non-clinical populations remain limited and require further well-controlled studies.

The mesolimbic pathway, involving projections from the ventral tegmental area (VTA) to the nucleus accumbens, is primarily responsible for reward processing and motivation. Its activation supports reinforcement-based learning, the subjective experience of pleasure, and the consolidation of goal-directed behaviour [23]. At the same time, dysregulation of this pathway has been linked to pathological motivation states, such as addiction, as well as to anhedonia and depressive symptoms, although effect sizes and replicability remain limited and strongly influenced by individual genetic and environmental variability [24,25].

Also, the mesocortical pathway, which exits the VTA and projects to the prefrontal cortex, is involved in the regulation of executive functions including planning, cognitive control, and working memory. Dopaminergic modulation within this pathway supports flexible cognitive performance, although neuroimaging and pharmacological studies suggest that these effects are of moderate magnitude and are strongly modulated by individual differences in dopaminergic gene polymorphisms, limiting the consistency of findings across studies [18,26]. Alterations in mesocortical dopaminergic signalling have been described in neuropsychiatric conditions such as attention deficit hyperactivity disorder and schizophrenia, although the phenotypic heterogeneity of these disorders complicates the isolation of specific dopaminergic mechanisms.

The tuberoinfundibular pathway regulates prolactin secretion and appears to have marginal relevance for sport and esports performance [27]. While its physiological role is well established, its potential influence on cognitive or behavioural function, particularly in the context of long-term pharmacological modulation, remains insufficiently explored.

Each of these pathways is characterized by a distinct neuroanatomical architecture, specific receptor distributions, and unique regulatory mechanisms. This complexity allows dopamine to simultaneously support motor, cognitive, emotional, and endocrine functions. Understanding the interactions between these pathways and their individual modulation opens new perspectives in the study of human psychophysical functioning and in the analysis of an individual’s predisposition, both in the context of health and performance in sport and esports.

**Figure 1:**
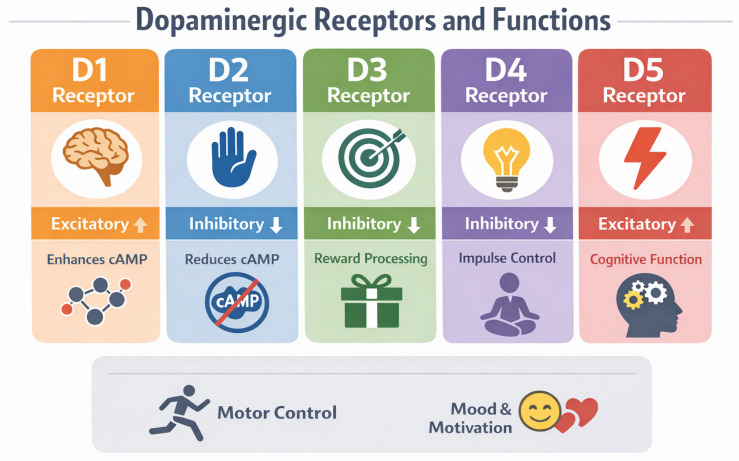
Schematic overview of dopamine D1–D5 receptors: family classification, main signalling pathways, and predominant neuroanatomical localisation.

Dopamine receptors comprise five subtypes (D1–D5) that belong to the family of metabotropic G protein-coupled receptors and are functionally divided into two main classes. D1-type receptors (D1 and D5), coupled to Gs proteins, stimulate adenylate cyclase activity and increase cyclic adenosine monophosphate (cAMP) levels in cells, while D2-type receptors (D2, D3, and D4), coupled to Gi/o proteins, inhibit adenylate cyclase and decrease cAMP production. These classes of receptors are widely distributed across brain regions such as the prefrontal cortex, striatum, and hippocampus, where they modulate neuronal excitability, synaptic plasticity, and cognitive functions, including executive control and working memory [28]. Functional differences between D1- and D2-type receptors contribute to the regulation of attentional stability, motivational salience, and behavioural flexibility [20,29]. The main distribution and signalling properties of D1–D5 receptors are summarised in Figure 1.

Although dopamine receptor subtypes that operate in distinct neuroanatomical and functional contexts, their combined action enables a fine-tuned modulation of reward-related behaviour and cognition, strongly dependent on environmental context and interactions with other neurotransmitter systems. Their detailed location and functional characteristics are shown in Table 1 [28,30].

**Figure 2:**
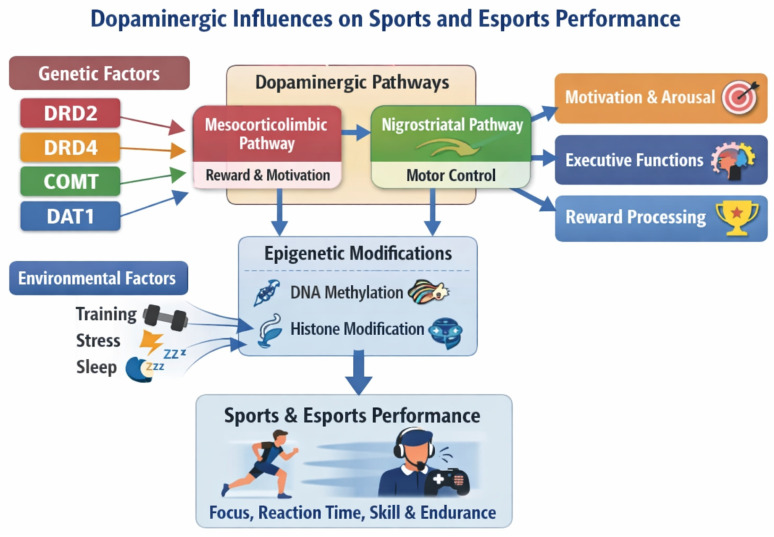
Conceptual framework illustrating the relationships between dopaminergic genetic factors (*DRD2*, *DRD4*, *COMT*, *DAT1*), major dopaminergic pathways, epigenetic mechanisms, environmental influences, and performance-related traits in sports and esports.

Dopamine is a key integrator of motor, cognitive, emotional, and endocrine functions, acting through diverse pathways and receptors distributed throughout the brain. The heterogeneity of its receptors—both in terms of structure and location—allows neuronal responses to be fine-tuned to changing environmental conditions and the body’s internal needs [31]. Understanding the function of individual dopaminergic proteins not only deepens our understanding of the neurobiology of behaviour but also opens new research opportunities for optimising performance in sport, esports, and everyday cognitive functioning. Figure 2 presents a conceptual overview integrating dopaminergic genetic factors, major neural pathways, epigenetic mechanisms, and environmental influences in relation to sports and esports performance.

## 4. Genes and Polymorphisms of the Dopaminergic System

The previous chapter described the molecular structure and distribution of dopamine receptors (D1–D5), while this section discusses the best-known polymorphisms in the genes encoding these receptors and, in the genes involved in dopamine synthesis, degradation, and transport. These variants are among the most frequently studied factors influencing individual differences in cognitive, motivational, and emotional functions, as well as susceptibility to various neuropsychiatric disorders [32].

Among the genes encoding receptors, *DRD2* and the dopamine D4 receptor gene (*DRD4*) are particularly noteworthy, as their functional variability is important for dopaminergic transmission. The *DRD2* gene encodes the D2 receptor, a key regulator of dopaminergic transmission in the striatum and prefrontal cortex. Polymorphisms such as rs1800497 and rs1076560 affect dopaminergic signalling and receptor availability [28,33,34]. The rs1800497 polymorphism, located in the neighbouring ankyrin repeat and kinase domain containing 1 gene (*ANKK1*) but linked to *DRD2*, has been associated with reduced D2 receptor availability and altered dopaminergic tone [34,35]. Allele A1 (T) correlates with lower D2 receptor binding potential in the striatum in healthy individuals, suggesting its influence on receptor density and dopamine feedback mechanisms [35]. However, the effects of this variant depend on the environmental and physiological context, including behavioural factors, which may explain the ambiguity of the research results [36].

Another frequently analysed variant, rs1076560, has been linked to differences in dopaminergic signalling and neural activation during cognitive tasks, although reported effects vary across studies and populations [37]. These findings emphasise that receptor polymorphisms rarely act in isolation and that their behavioural consequences depend on interactions with the environment, task demands and individual neurochemical background [26,38]. Overall, *DRD2* polymorphisms illustrate how dopaminergic genetic variants exert subtle, context-dependent effects rather than deterministic influences on behaviour. It is worth noting that a study published in the Baltic Journal of Health and Physical Activity, compared athletes and non-athletes and reported a higher prevalence of the Taq1D rs1800498 (*DRD2*) variant among athletes. Carriers of this variant also scored higher on the NEO Five Factor Inventory (NEO-FFI) extroversion and conscientiousness scales, suggesting that this polymorphism may be associated with personality traits beneficial to athletic performance [39].

The second gene of great research significance is *DRD4*, characterised by high allelic variability. The most frequently analysed variant is the 48 bp variable number tandem repeats (VNTR) in exon 3, which leads to the formation of receptor isoforms differing in the number of tandem repeats, most often 4-repeat (4R) and 7-repeat (7R) [40]. The 7R allele has been associated with reduced sensitivity to dopamine and altered intracellular signalling, which may influence behavioural plasticity and responsiveness to rewarding stimuli [41,42]. Carriers of this allele tend to show higher levels of novelty seeking and exploratory behaviour, although these effects are strongly modulated by environmental factors such as upbringing and stress in early life [43,44]. Consequently, the behavioural impact of the *DRD4* VNTR appears to be context-dependent, with the same variant potentially supporting either adaptive flexibility or risk-prone behaviour depending on environmental conditions. Another polymorphism of this gene, rs1800955, located in the promoter region, has been associated with differences in transcriptional activity and personality traits, although findings across studies remain inconsistent [45,46]. For example, research conducted in elite martial artists indicated associations between *DRD4* VNTR genotypes and selected NEO-FFI personality dimensions, but these effects varied across traits, and genotypic groups [47]. This pattern makes *DRD4* particularly relevant for disciplines requiring rapid adaptation and exploratory behaviour [48], although evidence in esports remains largely indirect. Overall, *DRD2* and *DRD4* illustrate the multigenic and context-dependent nature of dopaminergic modulation—individual variants have subtle but measurable effects on cognition and behaviour, which may be amplified in interaction with environmental or developmental factors. The most frequently studied polymorphisms of dopamine D1–D5 receptor genes (*DRD1–DRD5*), their location, and possible mechanisms of action are presented in Table 2.

In addition to receptor variability, dopamine levels also depend on genes involved in the synthesis, degradation, and transport of the neurotransmitter. The best known of these are catechol-O-methyltransferase (*COMT*), Solute Carrier Family 6 Member 3 (*SLC6A3*) (also known as *DAT1*) and *TH* [69,70,71]. The *COMT* gene, located on chromosome 22q11.21, encodes the enzyme catechol-O-methyltransferase, which is responsible for the degradation of catecholamines, including dopamine, in the prefrontal cortex. The functional polymorphism rs4680 results in a valine-to-methionine substitution (Val158Met) results in amino acid change that leads to reduce enzyme’s activity and consequently higher dopamine availability in the prefrontal cortex [72,73,74]. The Met allele has been associated with better performance in tasks requiring working memory and executive functions, as well as with inter individual differences in emotional and behavioural disorders [18,20]. Humińska-Lisowska et al. [75] analysed combat sports athletes and control subjects and demonstrated that the distribution of Val158Met genotypes differed between groups, and that the *COMT* genotype significantly interacted with personality traits such as novelty seeking, self-control, and self-transcendence [75]. These observations are consistent with the notion that balanced dopaminergic signalling in the prefrontal cortex is critical for optimal cognitive functioning.

The *SLC6A3* gene, located on chromosome 5p15.33, encodes the dopamine transporter (DAT), which is responsible for its reuptake and maintaining extracellular concentration balance. The most studied polymorphism is the 40 bp VNTR in the 3′ UTR region, where the 9-repeat (9R) and 10-repeat (10R) alleles are dominant. The 9R allele is associated with lower transporter expression and higher dopamine levels in the synaptic space, while 10R is associated with greater DAT affinity and more effective reuptake [76]. Neuroimaging and behavioural studies suggest that these variants influence reward sensitivity, attentional control, and impulsivity, although results remain heterogeneous across populations [77].

Another important element in dopamine synthesis is the *TH* gene, located on chromosome 11p15.5, which encodes tyrosine hydroxylase, an enzyme that limits the rate of conversion of tyrosine to L-3,4-dihydroxyphenylalanine (L-DOPA). Variability in the *TH* promoter region (e.g., rs10770141) can modulate transcriptional activity and the rate of dopamine production, and some studies link this polymorphism to differences in temperament traits and cognitive functioning in individuals with mental disorders [78,79]. Although less frequently studied than dopamine receptor genes or the *SLC6A3* transporter, *TH* variants may contribute to individual differences in baseline dopaminergic tone, particularly in interaction with stress or other genetic factors [80,81].

The collected data indicate that polymorphisms within dopaminergic genes act as subtle modulators rather than clear determinants of behaviour or cognitive functions. *DRD2* and *DRD4* variants influence receptor expression and sensitivity, while polymorphisms in *COMT*, *SLC6A3*, and *TH* regulate dopamine availability through metabolic and transport mechanisms. The combined effect of these genes shapes individual differences in motivation, cognitive flexibility, and emotion regulation. The most important polymorphisms of genes associated with dopamine metabolism and transport, their potential biological effects, and the research context are summarised in Table 3.

However, the functional effects of dopaminergic polymorphisms are strongly dependent on the environmental context. Stress, learning experiences and developmental conditions can modify the relationship between genotype and neural function efficiency, contributing to substantial inter-individual variability [14,91]. This dynamic interaction between genetic background and environmental factors help explain why the same variants may lead to different phenotypic outcomes across studies and supports the view that a multigene, network model of dopaminergic regulation is more appropriate than a ‘single gene’ approach.

## 5. Epigenetic Modulation of the Dopaminergic System in Athletes

There is increasing evidence suggesting that the variability of dopamine-dependent phenotypes is shaped not only by DNA sequence, but also by epigenetic mechanisms. This group includes DNA methylation, histone modifications, and the expression of non-coding RNAs, which regulate transcription without interfering with the nucleotide sequence itself. At the systemic level, regular physical activity and training loads generate reactive oxygen and nitrogen species, initiating changes in DNA methylation patterns and other epigenome modifications [92]. Reviews indicate that the response to a training programme depends on fitness status and that intensive training can be associated with telomere length changes and modulate DNA structure [93]. Methylation responses have been observed to vary depending on workload and sleep quality [94,95].

Epigenetics is also sensitive to psychosocial stress. A neuropsychological study has shown that methylation at the Val158 polymorphism site in the *COMT* gene is inversely correlated with the number of stressful life events and positively correlated with working memory performance. The results suggest that chronic stress may reduce methylation levels and increase *COMT* expression, leading to reduced dopamine availability in the prefrontal cortex. Methylation changes may therefore play a potentially compensatory role, possibly influencing the efficiency of executive networks [96].

Direct epigenetic evidence concerning athletes is still scarce, but pioneering studies have emerged in recent years. Humińska-Lisowska et al. analysed DNA methylation within the promoter and first exon of the dopamine transporter gene *SLC6A3* (*DAT1*) in athletes and physically inactive individuals. In athletes, a significantly higher methylation levels compared to controls, and the overall promoter methylation was markedly elevated. Personality assessment using the NEO-FFI questionnaire indicated that athletes scored higher on Extraversion and Conscientiousness, and promoter methylation levels were positively associated with selected personality traits. These findings suggest that increased methylation within the *SLC6A3* promoter region may be associated with training-related adaptations and may co-occur with personality characteristics conducive to athletic achievement [97].

Another advanced analysis was presented in a study of martial artists. The authors demonstrated that martial artists are characterised by higher methylation in the promoter region of *SLC6A3* and lower impulsivity than non-athletes. A significant interaction between the VNTR genotype and methylation was also found, even though allele frequencies did not differ between groups [98]. These results suggest that intensive training and competitive stress may induce adaptive epigenetic changes over time, confirming the plastic nature of methylation in athletes.

In healthy non-athletes, methylation of dopamine-related gene promoters—including *DBH* (Dopamine Beta-Hydroxylase), *SLC6A3*, and *DRD2*—was associated with performance on a line judgement task, and the combined methylation profile of these three genes explained a significant portion of the variability in attention lateralisation [99]. This means that epigenetic markers may be associated with not only personality traits, but also attention and perception processes.

In summary, epigenetic mechanisms play an important role in modulating dopamine availability in key areas of the brain. Unlike fixed polymorphisms, DNA methylation levels are sensitive to factors such as training loads, psychological stress, sleep, and diet. The integration of methylation measurements—especially in the promoter regions of *SLC6A3*, *COMT*, and *DRD2*—with behavioural assessments, training loads, and stress markers may allow for more precise modelling of psychomotor and motivational predispositions in sport and esport in future, provided that longitudinal designs and adequate control of confounding variables are applied.

## 6. Significance of Dopaminergic Gene Polymorphisms in the Context of Predisposition to Physical and Esports Activity

Contemporary research on sport and its digital variant, esports, emphasises that the success of competitors is determined by a complex and dynamic interaction of psychological, neurophysiological, and genetic factors [100]. A narrative review of the role of dopamine in sport indicates that, in addition to physical fitness and technical skills, mental resilience and the ability to cope with stress, which are shaped by both innate genetic predispositions and environmental influences, are of key importance. The authors estimate that approximately 66% of the variability in an athlete’s status may be attributed to genetic factors, with the remainder depending on training, diet, and life experiences. The same review emphasised that dopamine, a key neurotransmitter in the brain, regulates behaviour, motivation, learning, and motor control, pointing to a neurophysiological component of athletic success [3]. In addition, a meta-analysis of over 100 studies showed that psychological traits such as motivation, self-efficacy, conscientiousness, and extraversion have significant, albeit moderate, associations with athletic performance [101]. Taken together, these data confirm that performance in sport and esports is the result of a synergy between innate biological conditions and training and life experiences that shape an athlete’s temperament, mental resilience, and neurophysiology.

Esports introduce a separate dimension, which, although lacking a physical component, requires high cognitive skills, quick adaptation, and mental resilience. Research on League of Legends players shows that higher-ranked players are less extroverted and conciliatory, but more open to experiences than their colleagues from lower divisions [102]. Another cross-sectional study involving 416 esports players and 452 traditional athletes showed that esports players are less extroverted and less conscientious, although the number of years spent training in esports correlated positively with extroversion [103]. The authors suggest that the gaming environment may attract people with less need for social contact and lower levels of planning, while success favours those who are more cognitively flexible. The data available to date therefore points to a potentially specific personality profile in esports, different from that of traditional athletes; however, it is not known to what extent this is due to the same neurophysiological and genetic mechanisms that determine success in sport. This research gap becomes the starting point for further analysis in this chapter.

Dopamine is a key neurotransmitter involved in motivation, reward regulation, motor control, and executive functions [104]. In a narrative review on sport, Humińska-Lisowska describes how dopamine is sometimes referred to as the ‘motivation hormone for action and seeking new emotions’; this neurotransmitter affects a wide range of functions, from behaviour and learning to emotions and movement control, and differences in dopaminergic activity may translate into athletic predisposition and training perseverance [3]. At the genetic level, polymorphisms in the *DRD* gene, *COMT*, and *DAT1* are considered to play an important role. These variants modify receptor density, neurotransmitter degradation rate, and reuptake intensity, which influences individual differences in motivation, reward sensitivity, and stress resilience [3,105].

Most of the data comes from research on the *DRD2* gene. In an analysis of 258 men who practised combat sports and 284 non-exercising volunteers, the *DRD2* promoter polymorphism (rs1799732; del/ins) did not differ in frequency between the groups, but the interaction between participation in sport and genotype influenced temperament traits. Among athletes, deletion carriers scored lower on the reward dependence scale and higher on the self-direction scale, suggesting that this variant may be associated with greater autonomy and composure. The same group of athletes showed higher levels of self-discipline and cooperation than the control group, although the differences were subtle [106]. Further studies confirm that in karate, judo, boxing, and wrestling athletes, having specific *DRD2* alleles may correlate with personality traits such as conscientiousness and extroversion, which may promote regular training [107].

The analyses also focused on the rs6277 polymorphism and the rs1800497 variant associated with *DRD2* in individuals with problem gambling. In a study involving 168 students, genotyping showed that the combination of the T allele in rs6277 and the A1 allele in rs1800497, under conditions of high interpersonal stress, was associated with higher levels of problem gambling; this effect was mediated by stress avoidance strategies. The results suggest that the impact of stress on gambling behaviour may depend on genetic variants associated with *DRD2* and coping style, and that individuals carrying certain genetic profiles may be more likely to engage in gambling as a coping strategy when faced with social pressure [108]. However, it should be emphasised that the rs1800497 (Taq1A) variant is located in the neighbouring *ANKK1* gene, and its effect on the functioning of the dopamine D2 receptor is indirect, reflecting regulatory effects rather than a direct change in the *DRD2* coding sequence [109]. Although these studies did not strictly focus on professional esports players, they point to a potential gene-environment mechanism that may influence long-term engagement in video games.

The literature also discusses the involvement of other dopaminergic receptors. *DRD3* variants (e.g., rs167771) are associated with higher levels of agreeableness, which may facilitate cooperation in team sports [110], while polymorphisms in *DRD4* (e.g., variable number of VNTRs in exon 3) influence novelty seeking and impulsivity, which has been suggested to be associated with success in extreme sports [47]. However, these studies often have small sample sizes and are limited to selected disciplines, which makes it difficult to generalise the results. The *DRD1* and *DRD5* genes, which activate adenylate cyclase and increase cAMP production, have been studied less frequently, and a large Swedish cohort found only a slight correlation between the *DRD1* rs4532 variant and the level of moderate and intense physical activity in older people—CC genotype carriers were more active than T allele carriers [111].

In the context of dopamine-metabolising enzymes, particular importance is attached to the Val158Met (rs4680) polymorphism in the *COMT* gene, which affects the activity of the catechol-O-methyltransferase enzyme and dopamine concentration in the prefrontal cortex [112]. Studies of athletes suggest that carriers of the Met (A) allele are more resistant to stress and perform better in cognitive flexibility tests, although the results are inconsistent [113]. Polymorphisms in the *DAT1* transporter, especially the VNTR in the 3′ region, modulate the rate of dopamine reuptake; however, a large study of 8768 adults found no significant correlation between these variants and weekly exercise time, suggesting that single genetic markers have little predictive value in assessing exercise habits [114].

There is significantly less data available on electronic sports. Research on video game-related disorders indicates that *DRD2* polymorphisms may influence susceptibility to problematic gaming, especially in the presence of stress, but there is a lack of research on the population of professional esports players [108]. Neuropsychological data suggest that professional gamers may have a different personality and cognitive profile than athletes; greater openness and cognitive flexibility may compensate for a lower need for sensory stimulation, as reflected in the results of personality studies of gamers [115]. Potential studies could verify whether the *DRD2*, *DRD4*, or *COMT* variants play a similar role in shaping motivation and playing style in esports players as they do in traditional athletes.

To summarise the issues discussed so far and to highlight selected dopaminergic polymorphisms analysed in the context of psychological and sporting predispositions—their location, functional significance, and potential impact on behaviours relevant to esports (Table 4).

In summary, current evidence suggests that dopaminergic polymorphisms have a moderate, context-dependent influence on personality traits and behaviours related to sporting activity. In traditional sports, certain variants of *DRD2* and *DRD4* may promote motivation to train and perseverance, while others (*DRD3*, *COMT*) may enhance cooperation and mental resilience. In esports, where motivation and reward are different in nature, genetic-environmental interactions require further analysis. In the future, the integration of genetic research with the assessment of psychological and environmental traits may contribute to the development of more precise research frameworks, rather than direct performance prediction.

## 7. Conclusions and Further Research Direction

The accumulated literature suggests the importance of the dopaminergic system as an axis integrating cognitive, emotional, and motor components in the context of athletic performance. Nevertheless, the current state of knowledge reveals significant gaps that limit the possibility of formulating a coherent model of neurogenetic predispositions to psychophysical performance. Despite the growing number of studies analysing individual polymorphisms within genes such as *DRD2*, *DRD4*, *COMT* and *SLC6A3*, the vast majority of available data is based on correlational analyses conducted in isolation from other levels of biological organisation, such as neuronal activity or personality structure [3,124].

One of the main methodological barriers remains the lack of studies integrating genotype data with functional neuroimaging results and detailed assessments of temperament and personality traits. Existing approaches rarely consider the interactions between the neurobiological profile and the psychological construct, leading to fragmented knowledge and difficulties in interpreting the functional implications of specific genetic variants [125]. In particular, there is a lack of studies linking the presence of specific alleles to dopaminergic system activity measured by functional magnetic resonance imaging (fMRI), positron emission tomography (PET), or electroencephalography (EEG), and simultaneous measurement of stable temperamental traits in a biological-psychological model. This type of data triangulation could contribute to more nuanced explanatory models that take into account both innate traits and neuroplasticity dynamically shaped by the environment [126]. Recent empirical work has demonstrated the value of integrative approaches, showing that polygenic co-expression indices combined with PET imaging may better reflect dopamine synthesis capacity than single nucleotide polymorphism (SNP) analyses [127], while cumulative genetic indicators integrated with EEG measurements reveal task-demand interactions that escape the detection of single polymorphism studies [128]. These findings underscore the need to move from reductionist models to comprehensive frameworks that account for the complexity of gene-brain-behaviour relationships in sporting contexts. Most existing studies have focused on single polymorphisms or a limited number of SNPs, despite the polygenic nature of motivation, cognitive performance, and behavioral traits. Future research should therefore incorporate a broader range of genetic variants to allow the investigation of cumulative and interactive effects. In addition, many studies to date have relied on relatively small samples, predominantly of European ancestry, with women often underrepresented. Given the importance of population background in genetic association studies, future research should include larger, more diverse cohorts across different ancestries and sexes. Taken together, these limitations highlight the early and heterogeneous nature of the field and the need for more robust, integrative, and well-powered studies.

The second key area requiring intensified research is epigenetics, which offers the potential to explain phenotypic variability that is not captured in purely genotypic models. As noted in a narrative review by Humińska-Lisowska, combining genetic and epigenetic data is a key direction in research on the psychomotor predispositions of athletes [3]. While physical exercise and training induce extensive epigenomic changes in human skeletal muscle and blood, including alterations in DNA methylation patterns, histone modifications, and non-coding RNA expression [129,130]. There is a lack of in-depth analyses covering dopaminergic genes and the biochemical pathways involved in their expression. Recent whole-genome methylation studies in athletes have documented training-induced epigenetic remodelling in multiple physiological systems [131,132], and mechanistic reviews linked redox signalling to changes in DNA methylation during adaptation to exercise [92]. However, direct evidence for epigenetic regulation of *SLC6A3*, *DRD2*, *COMT* or other dopaminergic genes in athletic populations is still insufficient. This issue is particularly important in the context of environments that chronically expose individuals to stress, such as elite sport or esports competition, which are known to modulate gene activity through permanent epigenetic changes without interfering with the nucleotide sequence [93]. Emerging evidence from neuroscience shows that physical exercise can restore methylation-dependent mechanisms in stress-related brain regions and increase behavioural resilience [133], suggesting that the dopaminergic system could potentially be similarly modulated through epigenetic pathways during sports training and competition. Future studies should therefore include measurement of DNA methylation patterns, histone modifications, and microRNA expression within the dopaminergic axis, considering variables such as duration of stress exposure, training intensity, and sleep quality.

Finally, it is important to highlight the clear lack of research focusing on the population of esports players, whose workload is primarily cognitive and affective rather than somatic. Most genetic analyses are conducted in the context of traditional sports, limiting the ability to extrapolate the results to the digital competitive environment. Meanwhile, esports offer a unique opportunity to study the impact of dopaminergic processing on cognitive functions under conditions of high pace, time pressure, and decision-making complexity [134,135]. The latest meta-analysis data confirms that esports expertise is associated with measurable cognitive advantages, particularly in the domains of spatial cognition and attention [136], and empirical studies have linked cognitive flexibility and decision-making performance to ranking in multiplayer battle arena games [137]. Furthermore, emotional regulation and managing frustration-induced performance deterioration (tilt) are critical psychological competencies in esports that may be modulated by dopaminergic function [138,139]. Prolonged esports sessions induce objective cognitive fatigue with clear neurophysiological signatures, including pupil constriction and decreased executive function, which do not correlate with subjective reports of fatigue [140], pointing to complex interactions between dopaminergic tone, cognitive demands, and performance consistency. Despite these insights into the neurocognitive architecture of esports performance, there is currently lack of research systematically verifying the relationship between dopaminergic gene variants and measures of cognitive performance, attentional control, and emotional regulation in this population. Given that dopamine synthesis capacity predicts cognitive effort allocation and decision-making errors in demanding conditions [141,142], and functional connectivity within dopaminergic networks supports rapid attention switching and sensorimotor decisions in gamers [143], the study of genetic predispositions in esports represents a critical frontier for understanding the neurobiological basis of digital competition.

In summary, future research should move beyond one-dimensional models of single polymorphisms towards an integrative approach combining genetic, epigenetic, neurophysiological, and psychometric data [66]. In this field, multimodal datasets comprising polygenic or co-expression indices should be used instead of isolated SNPs, combine PET and fMRI with rigorous assessment of temperament and personality, use sufficiently large samples to enable replication, and support multidisciplinary teams with standardised protocols to reduce heterogeneity and improve mechanistic inference [128,144]. Only such a multidisciplinary perspective will bring us closer to a holistic understanding of the neurobiological basis of psychophysical performance, both in classical sports and in contemporary forms of digital competition. The convergence of molecular genetics, functional neuroimaging, epigenomics and psychological sciences may change our understanding of individual differences in athletic and cognitive performance, which may ultimately inform future research on talent development, training optimisation and individual differences in performance.

## Figures and Tables

**Table 1:** Characteristics of dopamine receptor families, their mechanisms of action, and location.

Receptor	Receptor Group	Neuroanatomical Location of the Receptor	Biological Functions	Biological Effect	Mechanism of Action	Effect on Adenylate Cyclase
*DRD1* (D1)	D1 (D1-like)	Cerebral cortex, striatum, hippocampus, olfactory bulb, limbic system	Learning, working memory, executive functions, motivation	Neuron excitation, increased activity	adenylyl cyclase → ↑ cAMP	↑ cAMP (stimulation)
*DRD2* (D2)	D2 (D2-like)	Striatum, substantia nigra, thalamus, hypothalamus, cortex	Motor coordination, motivation, sleep, hormonal regulation	Inhibition of neuronal activity, inhibitory effect	Inhibits adenylyl cyclase → ↓ cAMP	↓ cAMP (inhibition or no effect)
*DRD3* (D3)	D2 (D2-like)	Limbic system, nucleus accumbent, striatum, hippocampus	Regulation of reward-related behaviour and emotional responses	Inhibition of neuronal activity, inhibitory effect	Inhibits adenylyl cyclase	↓ cAMP (inhibition or no effect)
*DRD4* (D4)	D2 (D2-like)	Prefrontal cortex, amygdala, hippocampus, striatum	Attention, impulse control, exploratory behaviour	Inhibition of neuronal activity, inhibitory effect	Inhibits adenylyl cyclase	↓ cAMP (inhibition or no effect)
*DRD5* (D5)	D1 (D1-like)	Frontal cortex, hippocampus, and cingulate cortex	Cognitive functions similar to D1	Neuron excitation, increased activity	Stimulates adenylyl cyclase → ↑ cAMP	↑ cAMP (stimulation)

Source: own elaboration based on Beaulieu & Gainetdinov [28], and additional review sources [30].

**Table 2:** Overview of selected *DRD1–DRD5* gene polymorphisms: location, mechanism of action, and research context.

Gen	Polymorphism	Location (Genomic/ Structural)	Biological Functions	Biological Effect	Research
*DRD1*	rs4532 (A > G)	5′ UTR/promoter region	A nucleotide change may create short open reading frames that inhibit translation; potential impact on receptor expression.	It may modulate the effects of dopaminergic drugs and executive functions, but there is no clear clinical. evidence.	[49,50,51]
*DRD2*	rs1800497 (Taq1A, C > T)	Intron of the *ANKK1* gene adjacent to *DRD2*	The T allele (A1) reduces the density of D2 receptors in the striatum; it affects dopaminergic transmission.	Linked to differences in motivation, impulses and addictions, effect dependent on environmental context.	[34,35,36,52,53,54,55,56,57]
*DRD2*	rs1076560 (G > T)	Intron (affects splicing)	Allele T reduces the expression of the presynaptic short isoform of the dopamine D2 receptor (D2S), increasing the proportion of postsynaptic long isoform of the dopamine D2 receptor (D2L).	Associated with greater activity in the basal ganglia and prefrontal cortex during cognitive tasks, as well as with a higher risk of psychotic disorders.	[33,37,38,58,59,60]
*DRD2*	rs6277 (C957T, C > T)	Exon 7 (Synonymous polymorphism)	The T allele reduces mRNA stability but increases the availability of D2/3 receptors in the striatum.	Reduces the risk of schizophrenia in the European population, may affect working memory and learning through reinforcement.	[61,62,63]
*DRD2*	rs1799732 (-141C Ins/Del)	Promoter (Sp1 binding site)	The deletion reduces promoter activity in luciferase assays; it may alter receptor expression.	Variable effect on body weight and the action of neuroleptic drugs; stronger effect in women	[63,64]
*DRD3*	rs6280 (Ser9Gly, A > G)	Exon 1	The Gly allele increases the affinity of the D3 receptor for dopamine and enhances intracellular signalling.	May modulate response to reward and dopaminergic drugs, no clear effect on Schizophrenia.	[35,65,66,67]
*DRD4*	VNTR 48 bp (2R/4R/7R)	Exon 3 (tandem repeats)	Allele 7R reduces adenylate cyclase inhibition and dopamine sensitivity.	Increased impulsivity, novelty seeking; the effect depends on the environment and upbringing.	[40,43,44]
*DRD4*	rs1800955 (–521 C > T)	Promoter region (5′)	May affect promoter activity in neurons (no clear results)	Variability in personality traits (extroversion, impulsivity); effect dependent on gender and environment.	[45,46,48]
*DRD5*	(CA) microsatellite (148 bp)	Approximately 18 kb above the gene	It does not encode a protein but may be coupled with another regulatory variant.	A slight increase in the risk of Attention Deficit Hyperactivity Disorder (ADHD) (OR ≈ 1.24)—mainly in the inattentive and combined subtypes.	[68]

Source: own work based on Beaulieu & Gainetdinov, Gluskin & Mickey [28,34].

**Table 3:** Polymorphisms of *COMT*, *SLC6A3*, and *TH* genes: location, biological and functional activity.

Gen	Polymorphism	Location (Genomic/ Structural)	Biological Functions	Biological Effect	Research
*COMT*	rs4680 (Val158Met, G > A)	Exon 4; chromosome 22q11.21	Substitution of valine for methionine (Val→Met) reduces COMT enzyme activity by approximately 35–40%, increasing. dopamine availability in the prefrontal cortex.	The Met allele is associated with better working memory and executive functions, but greater reactivity to stress and emotional disturbances.	[18,20,72,73,74,75,82,83]
*COMT*	rs4633 (C > T)	Exon 3; chromosome 22q11.21	Synonymous polymorphism, does not change the amino acid, but affects mRNA stability and enzyme expression.	Usually coupled with rs4680; affects cognitive and emotional functions in a similar way to Val158Met.	[72]
*SLC6A3*	rs28363170 (3′-UTR VNTR, 40 bp, 9R/10R)	Region 3′-UTR; chromosome 5p15.33	The number of repeats modulates gene expression levels; the 9R allele is associated with lower expression and less DAT, while the 10R allele is associated with higher expression and more efficient dopamine uptake.	This variant is associated with differences in reward sensitivity, impulsivity, and activity of the basal ganglia and prefrontal cortex in motivational tasks; effects depend on population and environmental factors.	[70,76,77,84]
*SLC6A3*	rs27072 (A > G)	Region 3′ UTR; chromosome 5p15.33	The variant affects mRNA stability and dopamine transporter expression levels; it may be linked to the 3′-UTR VNTR (rs28363170).	Associated with differences in attention control and impulsivity; moderate effects, often modulated by environmental factors.	[85,86,87]
*TH*	rs10770141 (C > T)	Promoter; chromosome 11p15.5	Allele T increases promoter activity and *TH* transcription, increasing dopamine synthesis.	May increase susceptibility to stress and the risk of depression or schizophrenia; effect dependent on environment.	[82,88]
*TH*	rs6356 C-824T	Promoter; chromosome 11p15.5	The C > T change affects the binding of transcription factors and the mRNA level of the enzyme.	Combined with differences in temperament and cognitive functions in people with mental disorders.	[79,83,88,89,90]

**Table 4:** Functional characteristics of polymorphisms in *DRD1*, *DRD2*, *DRD3*, *DRD4*, *COMT*, *SLC6A3*, and *TH* genes and their potential relevance to cognitive traits and esports performance.

Gen	Polymorphism (Variant)	Location	Functional Effect	Population and Research Results	Potential Impact on Characteristics in Esports
*DRD2*	rs1799732 (–141C Ins/Del)	Promoter 5′	The deletion reduces promoter activity and the number of D2 receptors.	Polish MMA fighters (*n* = 85) vs. controls (*n* = 284). The deletion was associated with lower reward dependence and less harm avoidance [106,116]	Lower reward dependence may promote independence and perseverance in stressful situations; the player may continue playing despite the lack of immediate rewards.
*DRD2*	rs1800498 (Taq1D, C/T)	Intron 1	It is believed that the T allele reduces the density of D2 receptors.	Polish athletes (*n* = 159) vs. control group (*n* = 232). Taq1D (C/T) was associated with higher conscientiousness in athletes [39]	May favour planning, discipline and strategic organization; beneficial for training and in-game strategy.
*DRD2*	rs1079597 (Tag1B, G/A)	Intron 1	Allele reduces receptor expression.	Professional athletes (*n* = 163) vs. controls (*n* = 232). G/G genotype and G allele were more frequent in athletes; athletes scored higher on extraversion and conscientiousness [117]	Variation may shape extraversion and conscientiousness, affecting social interaction, motivation, and discipline in esports.
*DRD2*	rs6277 (C957T)	Exon 7	The T allele reduces mRNA stability and D2 expression.	Skiers and snowboarders (M = 341, F = 258); Caucasian; C allele increases D2 expression while T allele decreases it [118]	Reduced receptor availability may affect reward processing and reaction speed; potential influence on focus and motivation, though unstudied in esports.
*DRD2/ANKK1*	rs1800497 (Taq1A, A1/A2)	*ANKK1* gene (close to *DRD2*)	Allele A1 reduces the availability of D2 receptors.	Elite athletes (*n* = 60) vs. (*n* = 20) control group. Studies indicate the A1 allele is linked to increased impulsivity and risk-taking [119]	May promote risk-taking and reward-seeking behaviours; could be advantageous in high-risk gaming strategies, but evidence in esports is lacking.
*DRD3*	rs167771 (A/G)	Intron	The A/A genotype is associated with greater agreeableness; G reduces sensation seeking.	Football players carrying A/A genotype showed higher agreeableness compared with carriers of the G allele [110]	Enhanced agreeableness may support teamwork and communication; reduced sensation seeking could lower risk-taking, potentially leading to more conservative play.
*DRD3*	rs6280 (Ser9Gly)	Exon 1 (amino acid change)	The Gly allele increases receptor affinity.	No direct sports studies; functional assays indicate a higher affinity for dopamine for the Gly variant [114]	Increased receptor sensitivity may heighten reward responsiveness and motivation; implications for gaming performance remain speculative.
*DRD4*	VNTR (Number of repetitions)	Exon 3	Long alleles (≥7 repeats) reduce receptor binding and lower cAMP.	Meta-analyses show carriers of the long allele score higher on novelty-seeking traits [47,120,121]	Higher novelty seeking may foster exploration and creative strategies, but could also lead to impulsive risk-taking in esports.
*DRD5*	VNTR (130–166 bp)	Promoter	The 148 bp allele reduces D5 expression.	The 148 bp allele of the microsatellite near *DRD5* has been associated with ADHD, but not directly with receptor levels. Association studies indicate the 148 bp allele is linked to ADHD and decreased attention, but there are no athlete-specific data [122]	Reduced receptor function may diminish dopaminergic signalling and attention; possible impact on focus and reward processing in esports, but the evidence is indirect.
*COMT*	rs4680 (Val158Met, G/A)	Exon 4	The Met allele reduces COMT activity (↑dopamine)	Combat sports athletes (*n* = 258) vs. controls (*n* = 278). Athletes had different genotype frequencies and showed significant interactions between the *COMT* genotype and personality traits such as novelty seeking, self-management, and self-transcendence [75]	Lower *COMT* activity (Met allele) elevates dopamine, which may enhance cognitive control, stress resilience, and self-management-traits advantageous for sustained focus and emotion regulation in esports.
*SLC6A3* (*DAT1*)	VNTR (9R/10R)	3′UTR	The direction of the effect may depend on the study.	Polish male combat sports athletes (*n* = 200) Polish male controls (*n* = 102); 9/10 VNTR genotype associated with lower levels of anxiety in CS; 10/10 VNTR genotype associated with lower agreeability in CS [123]	10R allele carriers may exhibit greater aggressiveness and excitability, possibly favouring assertive play; 9R allele carriers may be more sensitive to reward and punishment, influencing risk assessment and emotional responses in competitive gaming.

## Data Availability

No new data were created or analyzed in this study. Data sharing is not applicable to this article.

## Abbreviations

The following abbreviations are used in this manuscript:

10R	10-repeat
4R	4-repeat
7R	7-repeat
9R	9-repeat
ADHD	Attention Deficit Hyperactivity Disorder
*ANKK1*	Ankyrin repeat and kinase domain containing 1
cAMP	Cyclic Adenosine Monophosphate
*COMT*	Catechol-O-methyltransferase
D1–D5	Dopamine receptor type 1–5
D2L	Long isoform of the dopamine D2 receptor
D2S	Short isoform of the dopamine D2 receptor
*DAT1*	Dopamine transporter
*DBH*	Dopamine Beta-Hydroxylase
*DRD1*	Dopamine D1 receptor gene
*DRD2*	Dopamine D2 receptor gene
*DRD3*	Dopamine D3 receptor gene
*DRD4*	Dopamine D4 receptor gene
*DRD5*	Dopamine D5 receptor gene
EEG	Electroencephalography
fMRI	Functional magnetic resonance imaging
L-DOPA	L-3,4-dihydroxyphenylalanine
NEO-FFI	NEO Five Factor Inventory
PET	Positron emission tomography
*SLC6A3*	Solute Carrier Family 6 Member 3
SN	Substantia Nigra
SNP	Single Nucleotide Polymorphism
TH	Tyrosine Hydroxylase
Val158Met	Valine-to-methionine substitution
VNTR	Variable Number Tandem Repeats
VTA	Ventral Tegmental Area
